# Maize plant detection using UAV-based RGB imaging and YOLOv5

**DOI:** 10.3389/fpls.2023.1274813

**Published:** 2024-01-04

**Authors:** Chenghao Lu, Emmanuel Nnadozie, Moritz Paul Camenzind, Yuncai Hu, Kang Yu

**Affiliations:** ^1^ Precision Agriculture Lab, School of Life Sciences, Technical University of Munich, Freising, Germany; ^2^ Mechatronics Research Group, University of Nigeria, Nsukka, Nigeria

**Keywords:** crop scouting, deep learning, plant detection model, UAV remote sensing, YOLOv5 application

## Abstract

In recent years, computer vision (CV) has made enormous progress and is providing great possibilities in analyzing images for object detection, especially with the application of machine learning (ML). Unmanned Aerial Vehicle (UAV) based high-resolution images allow to apply CV and ML methods for the detection of plants or their organs of interest. Thus, this study presents a practical workflow based on the You Only Look Once version 5 (YOLOv5) and UAV images to detect maize plants for counting their numbers in contrasting development stages, including the application of a semi-auto-labeling method based on the Segment Anything Model (SAM) to reduce the burden of labeling. Results showed that the trained model achieved a mean average precision (mAP@0.5) of 0.828 and 0.863 for the 3-leaf stage and 7-leaf stage, respectively. YOLOv5 achieved the best performance under the conditions of overgrown weeds, leaf occlusion, and blurry images, suggesting that YOLOv5 plays a practical role in obtaining excellent performance under realistic field conditions. Furthermore, introducing image-rotation augmentation and low noise weight enhanced model accuracy, with an increase of 0.024 and 0.016 mAP@0.5, respectively, compared to the original model of the 3-leaf stage. This work provides a practical reference for applying lightweight ML and deep learning methods to UAV images for automated object detection and characterization of plant growth under realistic environments.

## 1 Introduction

Maize is one of the most cultivated crops in the world and plays a vital role in food, fodder, and biomass fuel. Achieving high yield and quality in maize cultivation requires plant counting at early, mid, and late stages for various management strategies, including supplementary planting, pest management, and yield forecasting (Wang et al., 2023). Breeders examine the germination rate of new varieties in the field environment before selling them to the farmers to guarantee the quality of the seeds. Typically, germination rate is determined by manually counting plants in randomly selected blocks (Carciochi et al., 2019), which leads to a large uncertainty when counting plants manually due to the often large spatial variability (Rodríguez-Lizana et al., 2023). Furthermore, this method is time-consuming and therefore costly. Thus, it is necessary to develop a computer vision (CV) based approach for automatic counting of plants in the field.

The rapid advancements in CV and AI have led to efficient and accurate object detection and counting methods. For example, field robots and Unmanned Aerial Vehicles (UAVs) integrated with plant detection models are powerful tools for achieving automatic and accurate plant counting. However, field robots are only suitable for low-growing crops, such as the strawberry detection system of harvesting robot based on deep learning developed by Zhang et al. (2022). Furthermore, field robots encounter difficulty in movement in areas of high crop density, potentially leading to crop damage(Fountas et al., 2020). Given maize’s taller stature, UAVs are the preferred sensor carriers for its detection. With the rapid development of UAVs, UAV-based remote sensing has been widely used in precision agriculture, such as disease detection, growth monitoring, yield estimation, and weed management (Tsouros et al., 2019). Since UAVs can obtain high-resolution images and overcome the influence of cloud occlusion due to their low flight altitude, UAVs show enormous potential in collecting high-throughput phenotypic data.

Classical CV algorithms for object detection employ concrete-feature-based methods, mainly collecting features by manually integrating color, geometric, and texture features, and using non-neural methods for analysis (Rashed and Popescu, 2023). Valente et al. (2020) combined Vegetation Index (VI)-based vegetation classification with a plant average area filter to achieve plant counting, and showed that, in the experiment with a plant count of 170,000, the error rate was 42.5%. Such a high rate of errors indicates that traditional CV image processing techniques may not be able to overcome difficulties such as leaf occlusion and weed interference in plant counting (Shi et al., 2023). Recently, due to the continuous advancement and extensive use of deep learning, some abstract-feature-based methods have demonstrated excellent performance in detecting plants in complex field environments. Zhu et al. (2022) developed a wheat spike detection algorithm based on convolutional neural network (CNN) and transformer, achieving an average precision at a confidence threshold of 0.5 of 88.3%. Representative algorithms include CNNs and the You Only Look Once (YOLO) series of object detection models (Redmon et al., 2016). YOLO’s object detection mechanism is characterized by dividing images into a grid system, where each cell detects objects within it, enabling efficient and single-pass object detection. YOLO is known for its small model size and fast calculation speed (Bochkovskiy et al., 2020). YOLO is fast because it only needs one forward propagation to pass through the neural network to make predictions, and it only detects each object once (Srivastava et al., 2021). Given the advantages, the YOLO algorithm has been applied in a range of object detection applications requiring both simplicity and efficiency, particularly for plant detection tasks. For example, urban plantation tree detection with high-resolution remote sensing imagery based on YOLOv4-Lite (Zheng and Wu, 2022), real-time strawberry detection based on YOLOv4 (Zhang et al., 2022), crop diseases detection based on YOLOv5 (Zhao et al., 2023), and wheat spike detection in UAV images based on YOLOv5 (Zhao et al., 2021). Recently, variant versions of YOLOv5, notably the nano (n) and small (s) versions, referred to as YOLOv5n and YOLOv5s, respectively, have become attractive, considering the real-time performance requirements of YOLOv5 applied to UAVs or field robots. Nnadozie et al. (2023) compared the real-time performance of YOLOv5n and YOLOv5s on an NVDIA Jetson AGX Orin embedded GPU, finding that YOLOv5s and YOLOv5n achieved mAP@0.5scores of 0.924 and 0.904, respectively, under the conditions of image size of 640 x 480 and batch size of 8. As expected, the improvement in accuracy of YOLOv5s resulted in a loss of speed by 5FPS. However, this detection speed is still acceptable for the flight speed of UAVs, allowing UAVs to perform real-time detection. Li et al. (2022) also tested the YOLOv5s, m, l, and x models on 960 x 540 maize images, with mAP values of 87.65%, 90.24%, 91.02%, and 92.15%, respectively (Li et al., 2022). The average detection speeds were 54.9FPS, 49.3FPS, 44.6FPS, and 39.1FPS, while the model sizes were 14.1MB, 40.8MB, 89.2MB, and 166MB respectively (Li et al., 2022). Considering the future application of the model on UAVs, which requires accuracy, speed, and lightweight, this research will focus on investigating the YOLOv5s model for plant detection.

In the field of maize detection, many DL-based methods have also emerged. Kitano et al. (2019) used the U-Net of the CNN architecture to segment the green vegetation from the field, and then used the canopy area of a single maize plant for screening to realize the counting of maize. While such method often fails to distinguish between weeds of similar shape and size, and performed poorly when leaf occlusion existed, YOLOv5 has better performance in these two aspects (Li et al., 2022). Efforts have also been made to apply YOLOv4 and YOLOv5 to reduce the impact of weeds on maize counting (Mota-Delfin et al., 2022), though the mAP was only 77.6%; this indicates that there still room for improvement in the application of YOLOv5 for maize plant counting. So far, research on the application of YOLOv5 in different environments and phenological stages is still limited, such as research on leaf occlusion, weed interference, and phenotypic differences of plants in different growth periods. In addition, traditional model training requires extensive manual annotation for data labels, which is a complex and tedious task (Han et al., 2022). Exploring new annotation methods would be critical to fully realizing the potential of UAV imagery in field crop research. Segment Anything Model (SAM) is a segmentation model trained with over 1 billion masks from 11 million images (Kirillov et al., 2023), its performance rivals that of fully-supervised models, which can be used for object detection and achieve semi-automatic annotation without further training. Collectively, research is still needed to investigate how the SAM data augmentation can be used in combination with YOLOv5 to improve object detection model and its application in plant counting.

Data augmentation is used to enhance the training data set’s size and quality, allowing to develop better object detection models (Shorten and Khoshgoftaar, 2019). There are two types of data augmentation methods. The first type is single image processing, including image rotation, flipping, zooming, clipping, color transformation, Gaussian noise adding, and many more (Wang and Song, 2020). This method can significantly increase the quantity of training data but cannot generate complex training background beyond the original data. This will limit the model’s performance in predicting new data sets. Another type of data augmentation method is based on multiple image processing, which includes mixing up and mosaicking of images and therefore creates new artificial training data, and can specially improve the detection of small objects (Solawetz, 2020). These data augmentation algorithms enlarge the number of features of the data set and improve the model performance when combined with each other (Hao and Zhili, 2020). Smaller objects are less well detected than larger objects because the detectors usually extract features through aggregating pixels in convolutional layers (Li and Wu, 2022). Smaller objects result in fewer features leading to a worse prediction. Although the same problem occurs in the YOLO series, YOLOv5 took advantage of mosaic to overcome this problem to some extent (Redmon et al., 2016). Mosaic data augmentation combines training images in specific ratios, which allows for the model to learn how to identify objects at a smaller scale than average. It is also helpful in model training to significantly reduce the need for a large mini-batch size (Solawetz, 2020). In this context, it is interesting to know whether, by retaining the original data augmentation of the model, added processes of rotating and blurring images can further improve model accuracy.

Therefore, the objectives of this study were: (1) to train a lightweight, fast, and precise maize detection model based on YOLOv5s and UAV images; (2) to verify the robustness of the YOLOv5 model in environments with dense weeds at the 3-leaf stage and leaf occlusion at the 7-leaf stage; (3) to improve the model’s accuracy by merging a low-noise image dataset, and applying random rotation data augmentation; and (4) to propose a semi-automatic labeling process based on SAM to reduce the time and cost of manual labeling.

## 2 Materials and methods

### 2.1 Trial description and UAV images acquisition

**Figure 1:**
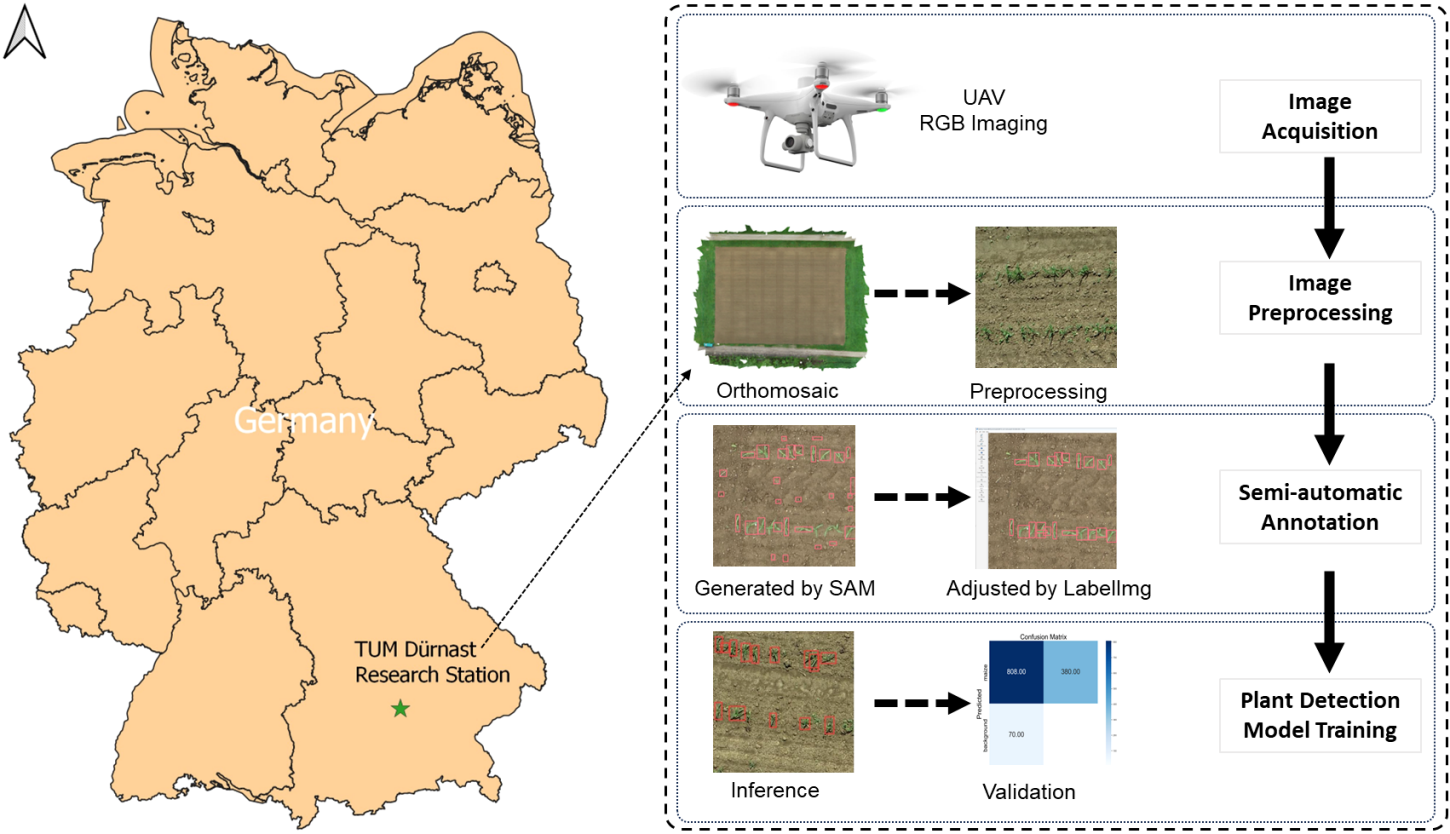
Study site and the workflow of maize plant detection.

The field experiment with maize plants was conducted in 2021 at the Dürnast Research Station of the Technical University of Munich in Germany (11.64323 E, 48.39749 N, **Figure 1**). Maize seeds were sown at the end of April with a density of 330 seeds m^-2^. A randomized complete block design with four replicates was used for the experiments. Plots consisted of 12 rows (1.5 m x 10 m).

The maize images were captured on the 11^th^ (3-leaf stage) and the 24^th^ (7-leaf stage) of June 2021, respectively. The UAV images of maize were taken by DJI P4 RTK (DJI, Shenzhen, China). The flight height was 12 m above ground level, and the flight speed was set to 2 m s^-1^. The ground sampling distance (GSD) is 0.33 cm/pixel. The images’ original size is 41075 x 37136 pixels, with an RGB color space of 24-bit depth, focal length of 8.8 mm and exposure time of 1/500 s, and the JPG format.

### 2.2 Image annotation

LabelImg (Tzutalin, 2015) is an image annotation tool developed based on Python (CWI, Netherlands). It can conveniently generate annotation files in YOLO format, which consists of object categories, the x-coordinate of the center point, the y-coordinate of the center point, the width ratio, and the height ratio. In LabelImg, a rectangle is drawn around the object of interest to create an annotation. The semi-automatic annotation process involves downloading the SAM model, adjusting the size range parameters of the mask, using SAM to detect the targets and generate annotations, and finally adjusting the label sizes and removing incorrect annotations in LabelImg. In this study we used the ViT-L SAM model. The parameter settings of the model can be seen in **Table 1**. SAM works by inputting images into the system, a grid of points is sampled over the image, SAM is applied to these points to generate initial masks, the quality of these masks is assessed using criteria like Intersection over Union (IOU) and stability scores, low-quality or duplicate masks are filtered out based on the assessment, additionally, a mask area filter is used to reduce the error masks, the final process concludes with the output of high-quality masks for the images and calculate the masks’ coordinates in YOLO format. Three datasets were used in this study, the 3l_origin dataset contains blurry images with a lot of weeds, while in the 3l_nonoise dataset, the images are clearer with almost no weeds. However, in the 7_origin dataset, severe leaf occlusion is present. Each dataset contains 200 512*512 pixels images that were cropped from the Orthomosaic. These datasets are annotated through SAM and manually adjusted through LabelImg.

**Table 1:** Parameter settings of SAM model.

Name	3-leaf stage	3-leaf stage with low noise	7-leaf stage
Points per side	32	32	32
Points per batch	64	64	64
Pred iou thresh	0.75	0.75	0.70
Stability score thresh	0.90	0.60	0.70
Stability score offset	1.0	1.0	1.0
Box nms thresh	0.3	0.3	0.3
Crop n layers	0	0	0
Crop nms thresh	0.7	0.7	0.7
Crop overlap ratio	512/1500	512/1500	512/1500
Crop n points downscale factor	1	1	1
Point grids	None	None	None
Min mask region area	40000	40000	40000
Output mode	Binary mask	Binary mask	Binary mask
Mask area filter	500 - 1500	200 - 1000	1500 - 5000

### 2.3 Model training workflow

**Figure 2:**
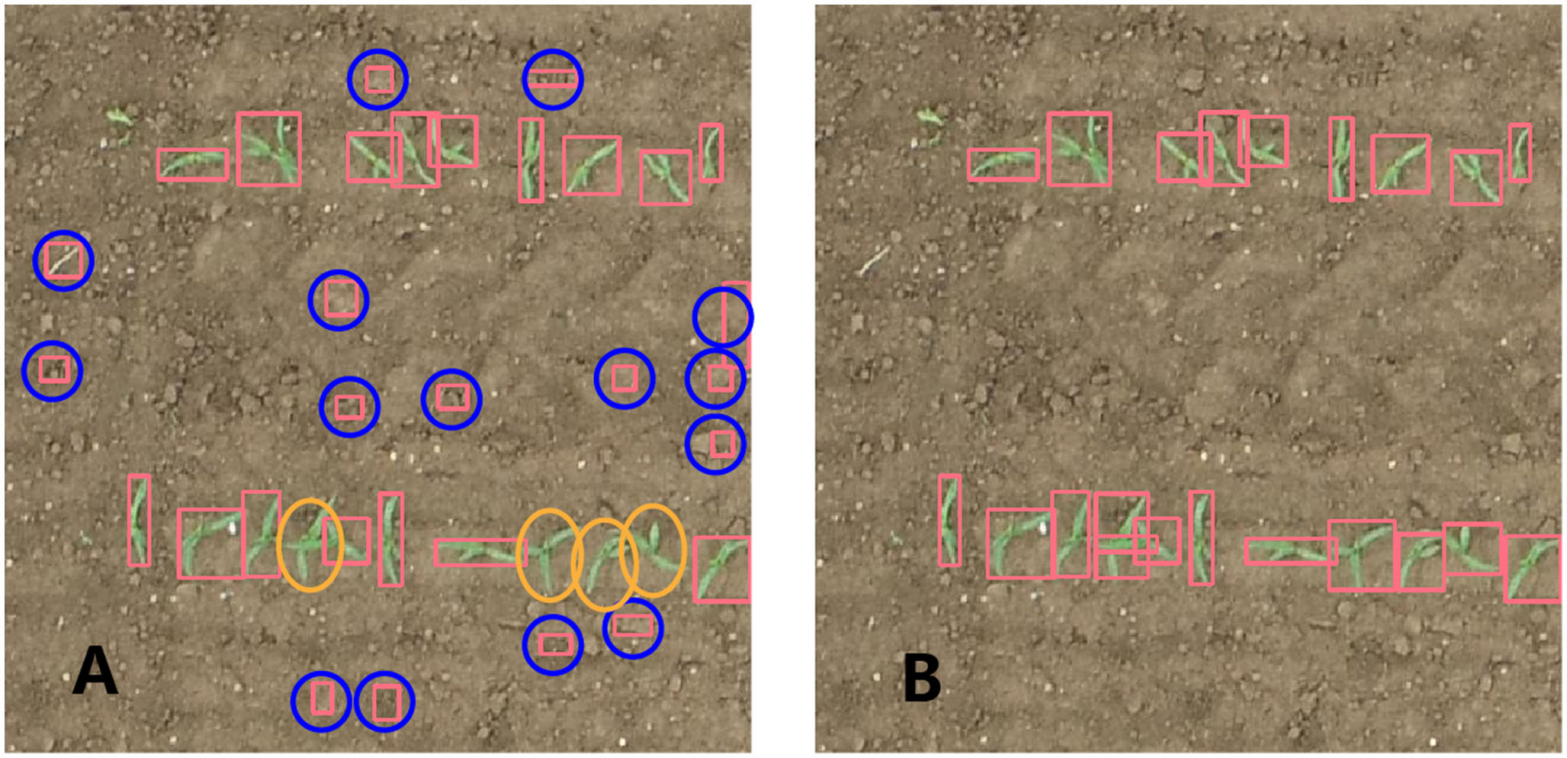
Image annotations: Generated by SAM **(A)** and adjusted manually by LabelImg **(B)**. The red rectangle represents maize seedlings, the blue circle represents erroneous identification by SAM, and the orange circle represents missed maize seedlings by SAM.

The workflow consists of image acquisition, data preprocessing, annotation generation, annotation adjustment, network training, and model testing (**Figure 1**). In the first step, we performed preprocessing of the images, including image filtering and cropping, to improve the efficiency of the model training. The images were labeled by SAM automatically for model training, using LabelImg to adjust annotations (**Figure 2**). Within LabelImg, incorrect SAM-generated labels were manually removed, and further adjustments were made to inappropriate annotations. Regarding the non-detected maize, rectangles were used to bind them and labeled them as “maize”. Subsequently, 200 annotated images were allocated as 70% for training, 20% for validation, and 10% for testing. We trained an original model (3l_origin and 7l_origin) with training and validation images. Considering that the images acquired by the UAV have different orientations, we applied a random rotation data augmentation of 90 degrees based on the original images; this augmentation rotated the image randomly between 0 and 90 degrees before the mosaic augmentation was applied. Following this, a model was trained with rotation augmentation (3l_90d and 7l_90d) and was used as pre-training weights to train the original dataset model (3l_origin_90d and 7l_origin_90d), to evaluate the impact of image rotation on model accuracy, in comparison with the original model. In addition, a nearly weed-free 3-leaf stage dataset (3l_nonoise) was created, and investigated the impact of incorporating this dataset on the original model (3l_origin_nonoise). Descriptions of all models in this study can be found in **Table 2**. The performance of YOLOv5 was tested in the case of severe leaf occlusion as well. The model training was performed on Google Colab equipped with a graphics processing unit (GPU) of Tesla K80 and 11441MiB memory. Training and validation data were fed into the YOLOv5s network to generate a model with a batch size of 9 and 400 epochs. Inference tests were subsequently conducted on the test dataset.

**Table 2:** The description of the models.

Model name	Description
3l_origin	This model was trained from the 3-leaf stage data set; there was no rotation data augmentation and low-noise images.
3l_90d	This model was trained from the 3-leaf stage data set, but the training set used 90-degree rotation data augmentation, with each image being randomly rotated within the range of 0 to 90 degrees.
3l_origin_90d	This model used the 3l_90d model as the pre-training weight and was trained from the 3-leaf stage data set, there was no rotation data augmentation and low-noise images.
3l_nonoise	This model was trained from a new low-noise 3l-stage data set. The images in this data set have higher definitions and fewer weeds.
3l_origin_nonoise	This model used the 3l_nonoise model as the pre-training weight and was trained from the 3-leaf stage data set, there was no rotation data augmentation and low-noise images.
7l_origin	This model was trained from the 7-leaf stage data set with no rotation data augmentation and low-noise images.
7l_90d	This model was trained from the 7-leaf stage data set, but the training set used 90-degree rotation data augmentation, with each image being randomly rotated within the range of 0 to 90 degrees.
7l_origin_90d	This model used the 7l_90d model as the pre-training weight and was trained from the 7-leaf stage data set, there was no rotation data augmentation and low-noise images.

### 2.4 Model performance evaluation

The performance of the models was tested on the validation set. A model prediction can be classified into four prediction cases, i) true positive (TP), ii) false positive (FP), ii) true negative (TN), and iv) false negative (FN) (Bochkovskiy et al., 2020). TP means the model detected the ground truth; FP represents that the model detected the incorrect object; FN represents undetected maize, and TN refers to objects of other categories correctly predicted by the model, because there is only one category; no other categories would be detected, TN is irrelevant in this work. Intersection over Union (IoU) is the ratio of intersection and union of the prediction box and ground truth box. It determines the prediction case and evaluates the distance between the predicted box and the target box (Zhang et al., 2022). An IoU of greater than 0.5 corresponds to TP, otherwise, to FN.

Precision (P) and recall (R) are defined as:


$$P=\;\frac{TP}{FP+TP}$$

$$R=\;\frac{TP}{FN+TP}$$

where P is the proportion of true positives in the detected maize. R is the ratio of the number of correctly detected maize to the total number of maize plants in the data set. P and R are usually negatively correlated. Therefore, the average precision (AP) was introduced to indicate the detection accuracy. AP refers to the average precision of the maize detection in the recall range of 0 to 1 (Zhao et al., 2021). A higher AP means a higher accuracy of the network. Following is the formula:


$$AP=\;\int_{0}^{1}P\left(R\right)dR$$

While mean average precision is the average AP of each class. The following is the formula:


$$\text{mAP}=\;\frac{1}{\text{N}}\int_{0}^{1}\text{P}\left(\text{R}\right)\text{dR}$$

Since there is only one class of detection objects in this study, AP is equal to mAP. In YOLOv5, mAP@0.5 and mAP@0.5:0.95 are two model evaluation indicators, meaning that when the IoU threshold is 0.5, the area under the smoothed P-R curve is calculated by integration as the final AP value, while mAP@0.5:0.95 refers to the average mAP of IoU from 0.5 to 0.95.

The Loss function consists of two components: the object loss (obj_loss) and the bounding box loss (box_loss). Object loss is used to supervise whether an object is present in the grid. Bounding box loss measures the difference between the predicted bounding box and the ground truth bounding box, which helps ensure that the model can accurately locate the object.

## 3 Results

### 3.1 UAV images and image annotation

**Figure 3:**
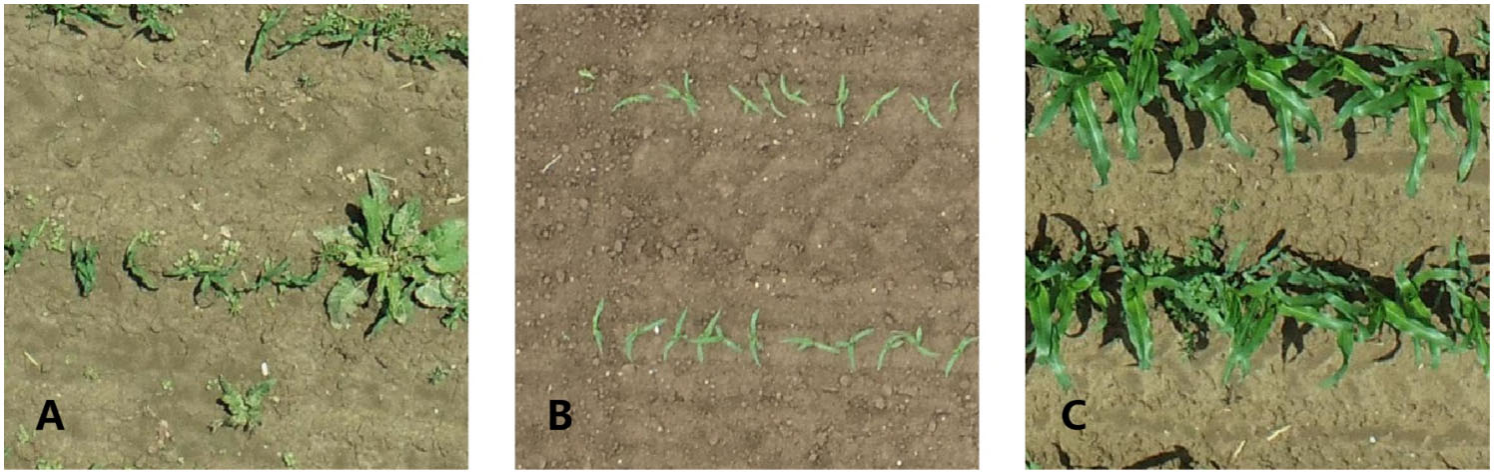
Acquired UAV Images: 3-leaf stage **(A)**, 3-leaf stage with low noise **(B)**, and 7-leaf stage **(C)**.

The images in the 3-leaf stage contained different sizes and shapes of weeds (**Figure 3A**), which might be similar to maize seedlings in their occurrence, and were used to distinguish between maize seedlings and weeds by YOLOv5. **Figure 3B** shows that there were almost no weeds in the images of the 3-leaf stage with low noise and minimal plant overlapping. The maize leaves in the 7-leaf stage were highly overlapping (**Figure 3C**), leading to difficulty in identifying each maize plant even by eyes. Thus, the images from the 7-leaf stage were used to test the detection ability of YOLOv5 under non-optimal conditions. **Figure 2A** displays the image annotations at the 3-leaf stage, which were generated by the SAM. **Figure 2B** demonstrates the manually adjusted results. SAM was used to annotate 140 images in each dataset and the inference speed reached 0.267 FPS. **Table 3** presents the inference results of SAM in three datasets. In the dataset of 3-leaf stage, there were a total of 1321 Ground Truth annotations. SAM generated 2992 annotations, out of which 1008 were correctly labeled as TP, 313 were labeled as FN and needed to be added, and 1671 were labeled as FP and needed to be removed. In the dataset of 3-leaf stage with low noise, there were a total of 2626 Ground Truth annotations. SAM generated 3781 annotations, out of which 2190 were correctly labeled as TP, 436 were labeled as FN and needed to be added, and 1165 were labeled as FP and needed to be removed. In the 7-leaf stage, there were a total of 1625 Ground Truth annotations. SAM correctly labeled 933 plants as TP, 692 plants were labeled as FN and needed to be added, and 1275 were labeled as FP and needed to be removed. Overall, 14.9% of the target annotations generated by SAM needed to be added, and 42.5% of the annotations needed to be removed.

**Table 3:** SAM inference results.

Results	3-leaf stage	3-leaf stage with low noise	7-leaf stage
Ground truth	1321	2626	1625
True positive	1008	2190	933
False negative	313	436	692
False positive	1671	1165	1275
Total detected	2992	3781	2890
Precision	37.6%	65.3%	42.3%
Recall	76.3%	83.4%	57.4%

### 3.2 Performance of different models

#### 3.2.1 Performance of training model

**Figure 4:**
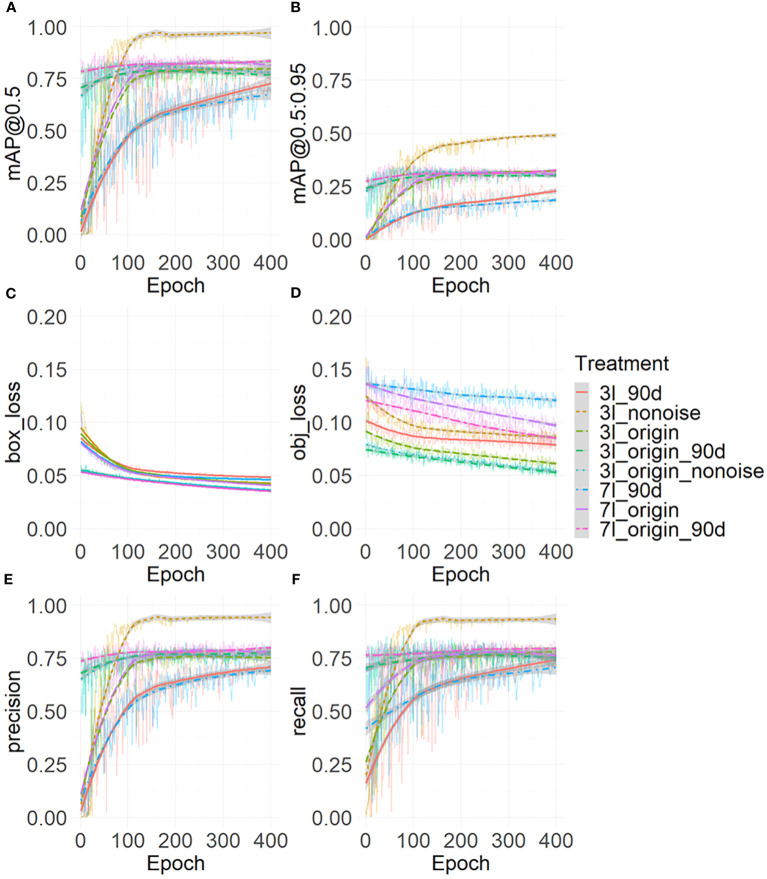
Model evaluation metrics: the object loss **(A)**, the bounding box loss **(B)**, the Precision **(C)**, the Recall **(D)**, the mAP@0.5 **(E)**, and the mAP@0.5:0.95 **(F)**. The thin line represents raw data, while the thick curve represents the results after local weighted regression scatter smoothing.

**Figure 4** shows the trend changes of object loss (A) and the bounding box loss (B), Precision (C), Recall (D), mAP@0.5(E), and mAP@0.5: 0.95 (F). These metrics increased with the iterations of epochs and began to plateau after reaching 130 epochs. During these 400 epochs, the best model was retained and further evaluated.

**Figure 5:**
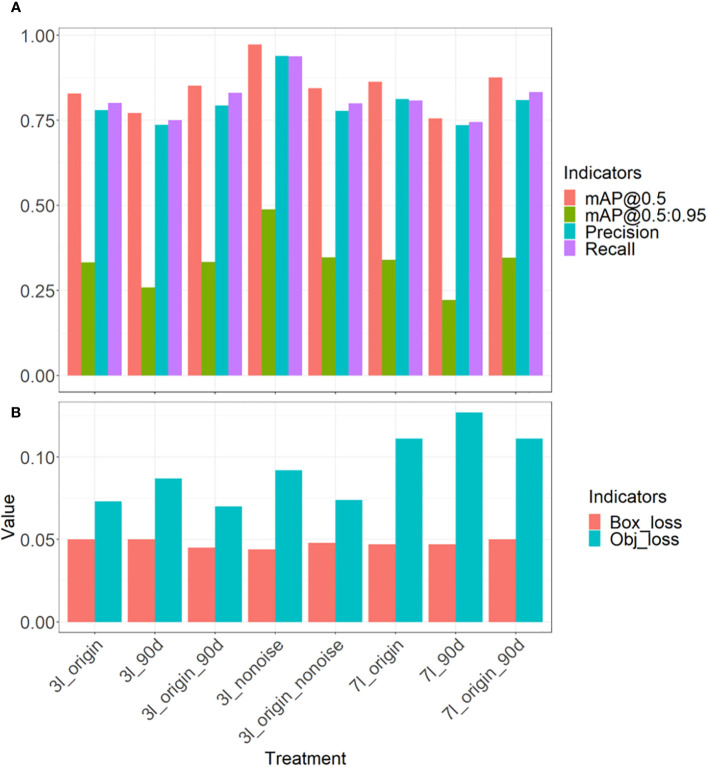
Comparison of the final performance of the models: Comparison of accuracy, including mAP, Precision, and Recall **(A)** and comparison of loss **(B)**.

**Table 4** and **Figure 5** show the final training performance of the model. The mAP@0.5of all models exceeded 0.75. When the models of the 3-leaf stage and 7-leaf stage were trained in this study, the leaf stage-specific models did not detect some maize of specific orientations. To understand the influence of rotation, the model at 90-degree angles was tested. The mAP@0.5of 3l_origin is 0.828, the lowest for 3l_90d is 0.772, and the highest for 3l_origin_90d was 0.833 (**Table 4**). In the models for the 7-leaf stage, the mAP@0.5 of 7l_origin was 0.863, the weakest for 7l_90d was 0.756, and the highest for 7l_origin_90d was 0.876. The rotation data augmentation improved the mAP@0.5 of the model by 2.4% at the 3-leaf stage and by 1.3% at the 7-leaf stage. Due to the presence of weeds and ambiguity in the original data set at the 3-leaf stage, many ambiguous labels were generated. We suspect that these labels might lead to an underestimation of the model’s ability, and thus, the 3l_nonoise model was trained on a low-noise data set, achieving a mAP@0.5 of 0.939. To explore whether low-noise weights can help the model work better under the influence of weeds and ambiguity, the 3l_origin_nonoise model was trained to achieve a mAP@0.5 of 0.852, which increased the mAP@0.5 of the model by 1.6% at the 3-leaf stage. Overall, rotation-based data augmentation showed more significant improvement than the low-noise data augmentation at the 3-leaf stage.

**Table 4:** The best training performance of the model.

Treatment	Obj_loss	Box_loss	Precision	Recall	mAP@0.5	mAP@0.5:0.95
3l_origin	0.073	0.050	0.780	0.801	0.828	0.333
3l_90d	0.087	0.050	0.737	0.751	0.772	0.259
3l_origin_90d	0.070	0.045	0.794	0.830	0.852	0.334
3l_nonoise	0.092	0.044	0.939	0.938	0.973	0.488
3l_origin_nonoise	0.074	0.048	0.778	0.833	0.844	0.347
7l_origin	0.111	0.047	0.813	0.808	0.863	0.340
7l_90d	0.127	0.047	0.736	0.745	0.756	0.222
7l_origin_90d	0.111	0.050	0.809	0.833	0.876	0.346

#### 3.2.2 Inference performance

**Figure 6:**
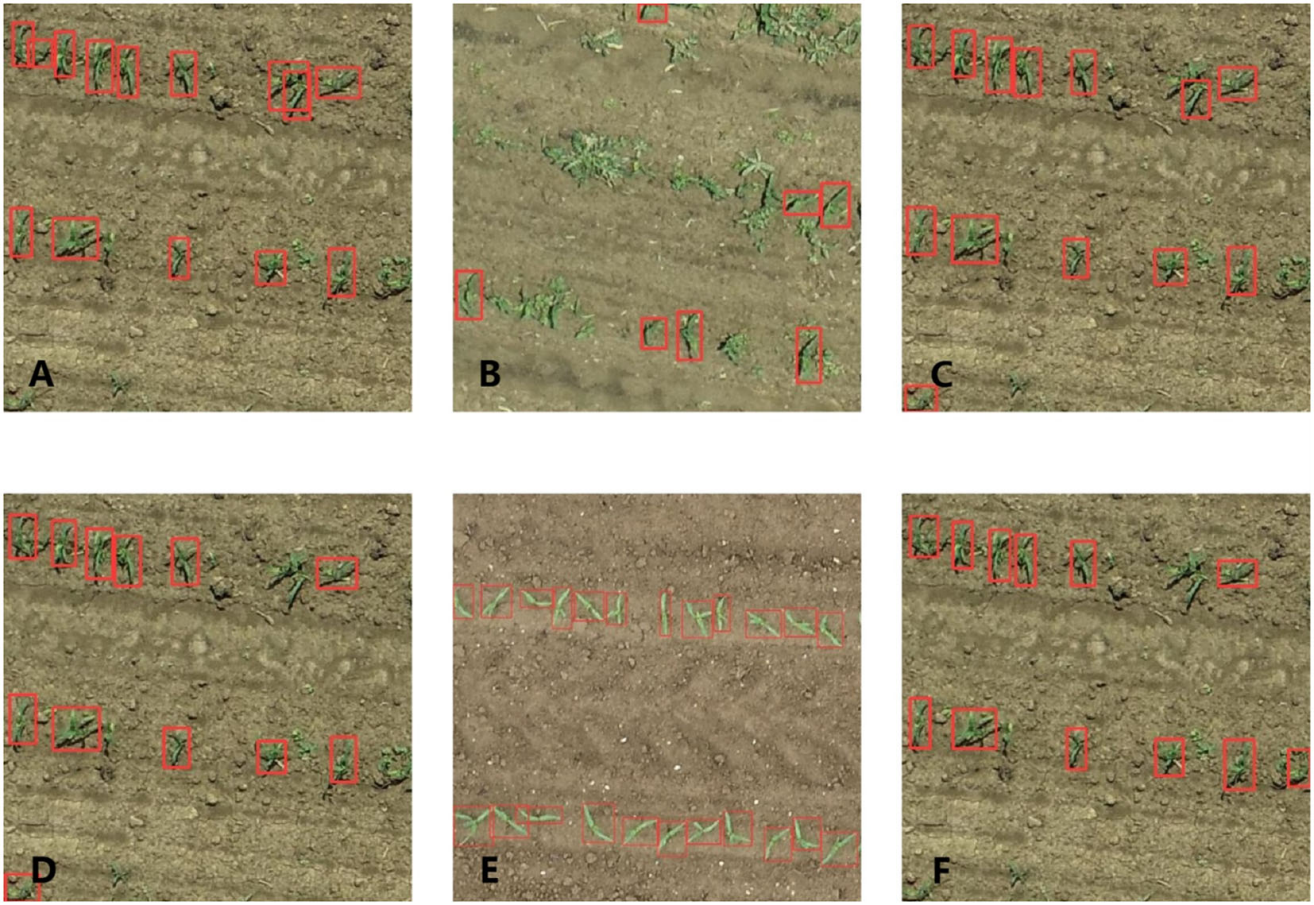
Practical performance of the 3-leaf stage model: origin model **(A)**, origin model in overgrown weeds conditions **(B)**; 90-degree rotation model in normal conditions **(C)**, origin model with a pre-training weight of 90-degree rotation model **(D)** low noise model in normal conditions **(E)**, and origin model with a pre-training weight of low noise model **(F)**.

**Figure 6** shows examples of using the 3-leaf stage model to predict a new dataset. **Figure 6A** presents the performance of the 3-leaf stage model without rotation in identifying maize in normal conditions; all 14 maize plants were detected without any incorrect results. **Figure 6B** shows the performance under overgrown weeds conditions; most maize plants were detected, and 4 maize plants were missed because of weeds clustering. **Figure 6C** is the performance of the 90-degree rotation model, 11 out of 14 maize seedlings were detected, and one incomplete maize seedling located at the edge of the image was also detected. **Figure 6D** is the original 3-leaf stage model trained based on the pre-training weight of the 90-degree rotation model; this means that the data set used in this model was entirely identical to that used in the original model, thereby allowing a fairer assessment of the impact of rotational data augmentation. **Figure 6E** is trained by a low-noise data set. **Figure 6F** is trained based on the pre-training weight of the low-noise model.

**Figure 7:**
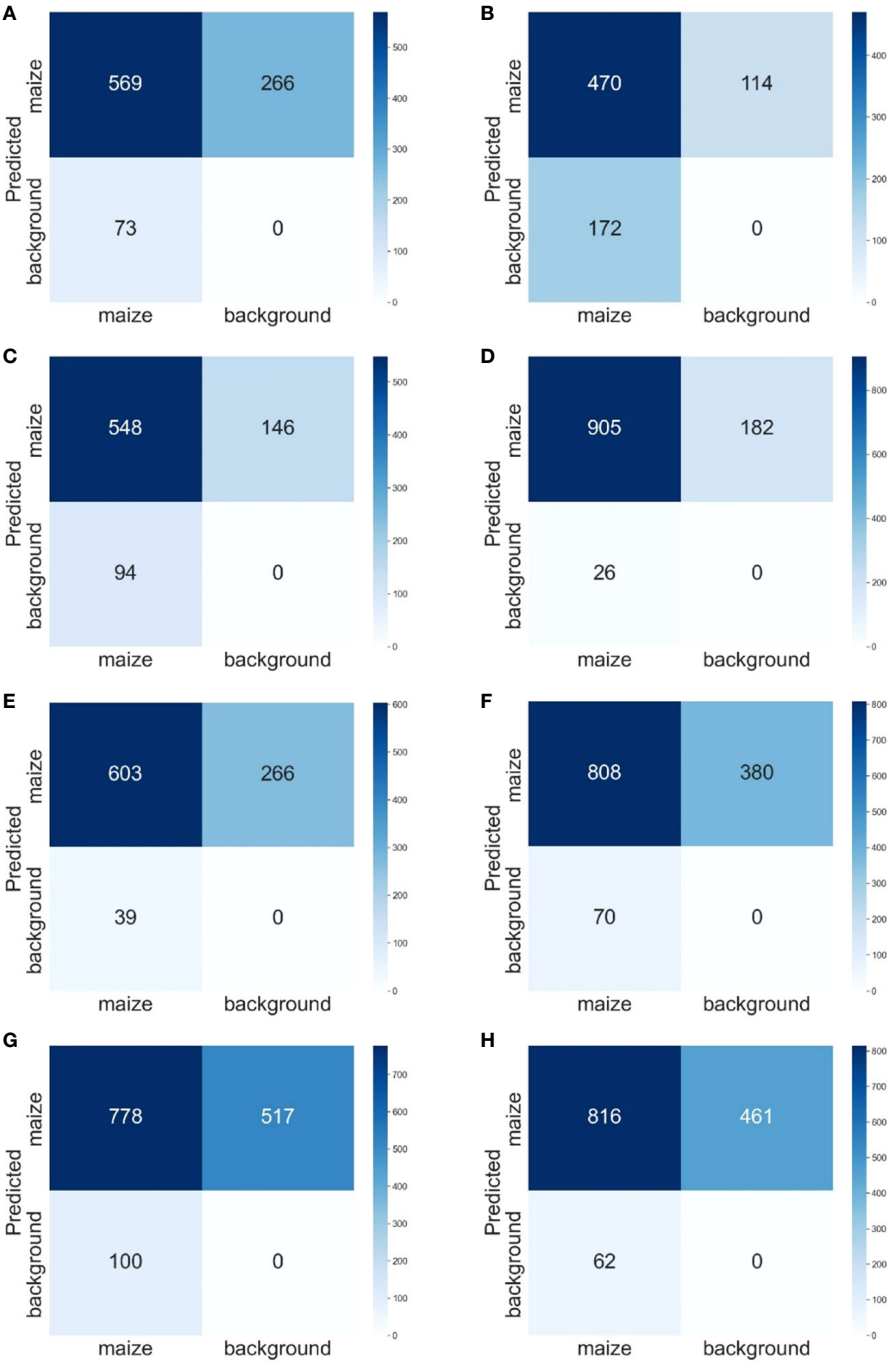
Confusion Matrix: 3l_origin **(A)**, 3l_90d **(B)**, 3l_origin_90d **(C)**, 3l_nonoise **(D)**, 3l_origin_nonoise **(E)**, 7l_origin **(F)**, and 7l_90d **(G)**. 7l_origin_90d **(H)**. The rows represent the prediction results, the columns represent the ground truth, and the number in the grid is the number of objects.

**Figure 7** shows the confusion matrix of each model. Comparing the confusion matrices of the 3l_origin model (**Figure 7A**) with the 3l_origin_90d model (**Figure 7C**), there was a slight decrease in TP, a slight increase in FN, and a significant reduction in FP. In the 7l_origin_90d model, there was a slight increase in TP, a slight increase in FP, and a significant decrease in FN.

**Table 5** shows the inference speed of all models. The inference speed of all models exceeds 150FPS, which is more than 500 times the inference speed of SAM, with the size of the YOLOv5s being only 13.7MB.

**Table 5:** Model inference speed.

Models	Speed (FPS)
3l_origin	154
3l_90d	159
3l_origin_90d	152
3l_nonoise	154
3l_origin_nonoise	167
7l_origin	154
7l_90d	154
7l_origin_90d	156

## 4 Discussion

### 4.1 The influence of weeds and leaf occlusion

In this study, plant detection results varied significantly between the two growth stages, influenced by differing weed densities. In the 3-leaf stage of a low weed density, the model demonstrated a relatively good performance in maize detection. However, the weed was sometimes mistakenly detected as maize plants – false positive (FP). On the one hand, this misdetection might be caused by the fact that the shape and color characteristics of weeds in such a relatively young growth stage are very similar to those of maize seedlings, and most models are based on the shape and color characteristics to detect the maize plants. On the other hand, models trained with the low resolution of UAV images could have further weakened the characteristic differences between weeds and maize, resulting in false positive cases. Besides, there was still a proportion of maize seedlings that were ignored by the model, mainly due to the clustering of weeds (**Figures 3A**, **6B**). Similarly, Casuccio and Kotze (2022) used YOLOv5 and high-resolution RGB images to evaluate the planting quality of maize showed the same problem that also existed in their research, and about 40% of the missed detections were caused by the occlusion of adjacent weeds. Additionally, as a result of the high flying altitude of the UAV, the relatively large GSD (0.33 cm) added to the influence of weed and leaf occlusion, i.e., resulting in blurry images, which leads to the omission of maize seedlings in the detection. The same issue also exists in other models. Velumani et al. (2021) reported that based on Faster-RCNN at a lower image resolution (larger ground sampling distance), the trained model showed more FP cases and lower average precision of only 0.64. In this research, due to the limitations of the UAV image resolution, the influence of weeds, and the mutual occlusion of plants, the annotation of the target plants could be more accurate. Therefore, selecting IoU of 0.5 is more suitable for evaluating the models in this study.

**Figure 8:**
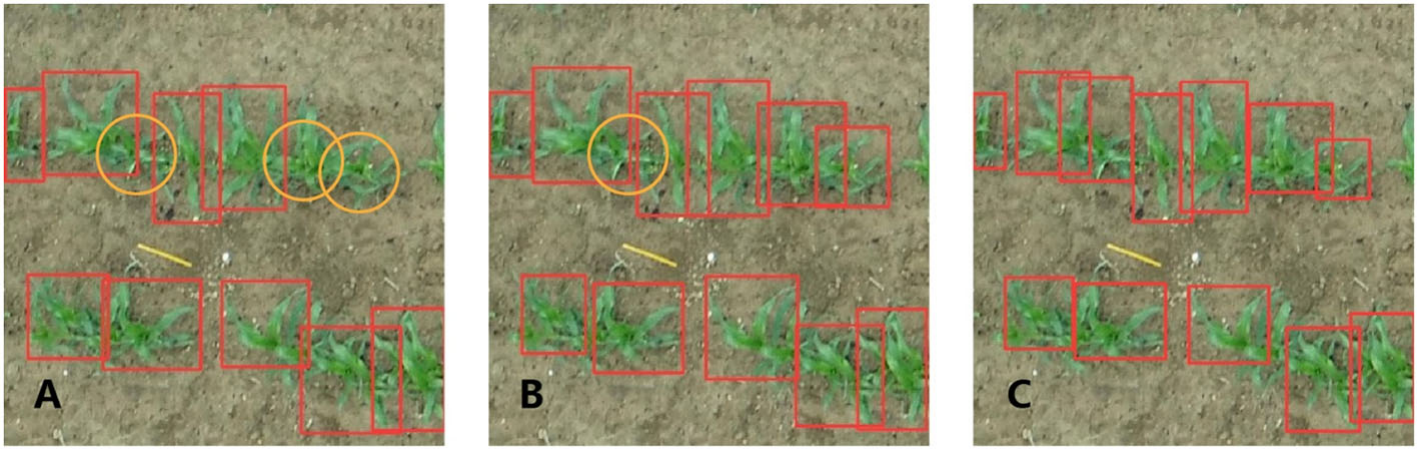
Practical performance of the 7-leaf stage model: origin model **(A)**, 90-degree rotation model **(B)**, and origin model with a pre-training weight of 90-degree rotation model **(C)**. The red rectangle represents the models’ predicted results, while the orange circle represents the maize plants that were missed by the model.

Although there was severe leaf occlusion in the 7-leaf stage, the model also performed relatively well in maize detection (**Figure 8**). The 7-leaf stage maize detection model yielded more FN cases in plant detection (**Figures 7F–H**). This might be associated with leaf occlusion because, in this growth stage, there is a significant difference in appearance between weeds and maize, resulting in fewer FP cases, i.e., weeds detected as plants. Nevertheless, severe leaf occlusion makes it challenging to collect training data and leads to rough annotation quality and model errors, eventually increasing FN cases. To address the problems of dense objects and complex background noise, Liu et al. (2023) proposed Feature Enhancement Block (FEBlock) and Self-Characteristic Expansion Plate (SCEP). This method could potentially improve our model accuracy for plant detection, which should be investigated in future work.

### 4.2 Improvement of model performance via augmentation

When the models of the 3-leaf stage and 7-leaf stage were trained in this study, some maize plants of certain orientations were not detected by the leaf stage-specific models with the application of rotation augmentation. The model with rotation data augmentation as pre-training weight was indeed able to detect some previously ignored targets, while it failed to detect some targets (**Figures 6A, D**). Thus, the rotation-augmented model increased the probability of correctly detecting the previously misidentified and undetected maize plants. In the 3l_origin_90d model, the significant decrease in FP might be due to the further intensified training of the model targeted for maize with different leaf orientations after the rotation, while the slight reduction of TP and a slight increase in FN may be due to the increased robustness of the model. With the changes in FP, TP, and FN, both Precision and Recall have been improved, resulting in a 2.4% increase in the mAP@0.5 of this model. The reason for the lowest mAP of 3l_90d is that the images in this dataset are cropped from a large-sized UAV image, resulting in a slight overfitting of the 3l_origin model. In contrast, in the 3l_90d model, the training set images were rotated while the validation set images were not, making it not meaningful to compare the two models. Therefore, the 3l_90d model was used as pre-training weights, and then the 3l_origin_90d model was trained on the same training set. In the 7l_origin_90d model, the increase in TP and decrease in FN might be attributed to the enhanced diversity of maize features in the model through rotation data augmentation, resulting in the detection of some previously omitted maize plants. Unfortunately, the FP of this model has increased, which might be due to the overlapping maize leaves and rotation augmentation could have caused the model to misjudge some overlapping leaves as maize. Another explanation would be that the model could have correctly detected corn, but the label was missing. Although there is a slight decrease in precision, there was a significant increase in recall, resulting in a 1.3% improvement in mAP@0.5 of this model.

Li et al. (2022) also developed a maize plant detection model based on YOLOv5. To improve the accuracy of YOLOv5, they focused on adjusting the model structure. In their work, the channel attention mechanism (SENet) was integrated into YOLOv5, and increased mAP by 1.21%. Therefore, it is worth noting that further work should investigate the extent to which different data augmentation and model structure adjustment methods can improve the accuracy of the plant detection model.

### 4.3 The challenge of labeling

Labeling was also challenging in this study, since the maize plants could not be distinguished from weed plants in the 3-leaf stage, especially those growing in the sowing row. In this stage, maize and weed were quite similar in their shapes and sizes, making the labeling - drawing a bounding box around an entire individual plant without including too much surrounding space, often impossible.

Usually, the weed plants between sowing rows could be easily detected. Weeds between rows usually have more surrounding space and less leaf occlusion, which is easily distinguishable. In contrast, some weed plants grow in the same clusters as maize plants, making them difficult to be labeled. In the 7-leaf stage, leaf occlusions between plants were quite severe. Therefore, counting the maize plants visually on the images is almost impossible. To improve the quality of labeling, labelers are advised to revise ambiguous annotations based on the actual situation in the field. Alternatively, obtaining a small GSD is recommended by using a higher pixel resolution camera or lowering the flight altitude. This would allow us to see more detail, thus improving the labeling and model training.

It is a common understanding that labeling is more time-consuming compared to model training. Based on SAM, a semi-auto-labeling method was proposed to help us reduce the labeling work in this study. Although most maize seedlings were accurately annotated, there were still some omissions and invalid annotations. Those omissions were mainly due to the inability of SAM to identify overlapping maize seedlings, which might have not yet fully developed the significant characteristics of an individual plant being learned by the model and thus ignored by the model (**Figure 2A**). Those invalid labels are also expected, because SAM will recognize all kinds of objects in the image. In this study, the background of maize is mainly soil, and some soil blocks were recognized by SAM. Although we only applied a size filter to filter some invalid annotations, adding a color filter probably can further reduce invalid annotations.

A straightforward question may arise, despite SAM’s ability to detect objects accurately, it’s a large model demanding substantial hardware resources, is slower in speed, and is challenging to adapt to complex field environments. Our goal for applications here was to train a lightweight model that can be applied to UAVs and field robots, and to propose the process of refining the rough large model into a delicate light model. However, substantial effort is still necessary to refine the annotations. Therefore, future work should continue to find out more efficient image labeling methods.

### 4.4 Future work

Model improvement in the future may consider multiple aspects. First, the number of training images should be increased to improve the model. Although only 200 images in this study were used to train the model, the model already showed considerable performance. Training should also incorporate images from diverse field conditions, including different crop varieties, soil backgrounds, and lighting conditions. To obtain the best data augmentation parameters for maize detection, it may be possible to use the hyperparameter evolution of YOLOv5 in the future. Furthermore, future work may also consider modifying the structure of YOLOv5 by adding a downsampling or up-sampling layer, changing the activation function, or trying other detection scales to better fit the maize plant detection (Zhao et al., 2021).

Previous comparisons of YOLOv5 with 15 other advanced UAV-compatible models revealed its superior speed, albeit with a slight accuracy trade-off (Zhan et al. (2022). Compared with the most accurate DPNet-ensemble model (Du et al., 2019), YOLOv5 improves the detection speed by eight times at 9.02% AP loss. These indicate that YOLOv5 has great potential in practical applications for being deployed on drones and field robots.

## 5 Conclusions

We proposed a maize plant detection method using semi-automatic annotation with SAM and YOLOv5 for analyzing UAV-based RGB images. With the data sets at 3-leaf and 7-leaf stages, models were separately trained to detect most maize plants under weed occurrence and leaf occlusion conditions. The model trained for the 3-leaf and 7-leaf stages reached an mAP@0.5 of 82.8% and 86.3%, respectively. Our study suggests that YOLOv5 based plant detection model shows the potential to be adaptable to various growth stages of maize plants. In addition, applying rotation-based data augmentation and low noise weight could improve robustness under realistic field conditions. The YOLOv5-based maize detection model shows promise for deployment on UAVs and other IoT devices for real-time plant monitoring.

## Data availability statement

The raw data supporting the conclusions of this article will be made available by the authors, without undue reservation.

## Author contributions

CL: Writing – original draft. EN: Writing – review & editing. MC: Writing – review & editing, Data curation. YH: Writing – review & editing. KY: Writing – review & editing.
